# High precision alignment of cryo-electron subtomograms through gradient-based parallel optimization

**DOI:** 10.1186/1752-0509-6-S1-S18

**Published:** 2012-07-16

**Authors:** Min Xu, Frank Alber

**Affiliations:** 1 Program in Molecular and Computational Biology, University of Southern California, Los Angeles, CA 90089, USA

## Abstract

**Background:**

Cryo-electron tomography emerges as an important component for structural system biology. It not only allows the structural characterization of macromolecular complexes, but also the detection of their cellular localizations in near living conditions. However, the method is hampered by low resolution, missing data and low signal-to-noise ratio (SNR). To overcome some of these difficulties and enhance the nominal resolution one can align and average a large set of subtomograms. Existing methods for obtaining the optimal alignments are mostly based on an exhaustive scanning of all but discrete relative rigid transformations (i.e. rotations and translations) of one subtomogram with respect to the other.

**Results:**

In this paper, we propose gradient-guided alignment methods based on two popular subtomogram similarity measures, a real space as well as a Fourier-space constrained score. We also propose a stochastic parallel refinement method that increases significantly the efficiency for the simultaneous refinement of a set of alignment candidates. We estimate that our stochastic parallel refinement is on average about 20 to 40 fold faster in comparison to the standard independent refinement approach. Results on simulated data of model complexes and experimental structures of protein complexes show that even for highly distorted subtomograms and with only a small number of very sparsely distributed initial alignment seeds, our combined methods can accurately recover true transformations with a substantially higher precision than the scanning based alignment methods.

**Conclusions:**

Our methods increase significantly the efficiency and accuracy for subtomogram alignments, which is a key factor for the systematic classification of macromolecular complexes in cryo-electron tomograms of whole cells.

## Introduction

Cryo-electron tomography emerges as an important component for structural system biology approaches [1,2]. Cryo-electron tomograms of whole cells essentially contain information on the systems level about the abundance, spatial distributions and orientations of all large macromolecular complexes at a given time point in a cell [3-9]. However, detecting these complexes in cryo-electron tomograms is a challenging task due to low signal-to-noise ratio (SNR), distortions and low non-isotropic resolution (> 4 nm) of the tomograms [6]. Therefore traditional image registration methods [10], developed for images at low distortion levels, usually cannot be directly applied to alignment of subtomograms. One strategy to enhance the nominal resolution of the detected density maps of individual complexes is to segment the tomogram into a large number of single complex subtomograms, which are then classified into similar objects by a pair-wise comparison. After subtomogram classification averaging of the aligned subtomograms in each class reveals the shapes of macromolecular complexes in each class at an increased SNR, which can then be assigned to the corresponding positions in the whole cell tomogram.

Subtomogram alignment and classification methods [6,11-26] are key to such processes and have been applied to several complexes, including membrane-bound complexes [27,28]. However, due to the potentially large number of subto-mograms in whole cell tomograms alignment protocols must not only be precise but also computationally efficient. Existing alignment methods are typically based on the exhaustive sampling over a discrete set of rigid transformations of one subtomogram with respect to a second. The optimal alignment is then detected using the dissimilarity measure between both subtomograms [11-13]. However, due to the heavy computational cost, the exhaustive rotational search can only sample a limited number of angles. Moreover the typically applied Fast Fourier Transform (FFT) based translational alignment can only approximate best translations at the resolution of the unit voxels. To enhance computational efficiency an approximate alignment method has been proposed to generate alignment candidates based on a fast translation-invariant rotational search [14,15]. Then a local refinement was used starting from the alignment candidates close to the optimal solution. However, the full potential of using only local refinements on very sparsely distributed starting candidates has not been investigated yet. In this paper, we propose an efficient gradient-guided alignment method based on two popular subtomogram dissimilarity scores. In addition, we design a stochastic parallel framework that significantly speeds up the simultaneous refinement of multiple alignment candidates.

We demonstrate on realistically simulated data of models and real macromolecular structures that for highly distorted subtomograms, even given a small number of evenly sampled initial angles with a large interval of 60° or 45°, our method can accurately recover true transformation with very high precision.

## Methods

Here we provide a gradient-guided refinement framework for subtomogram alignment that minimizes a dissimilarity score as defined by the squared sum of the differences between a parameter fixed function and a function whose parameters are optimized. We consider two types of dissimilarity scores for subtomogram alignments, which both incorporate missing wedge corrections: A real space constrained dissimilarity score (Section 2.2) and a Fourier space constrained dissimilarity score (Section 2.4). In addition, we adapt our refinement protocol also to the case where the rotational search is restricted to only certain axis of rotations, for instance when the search is constrained to rotations around a membrane surface normal when membrane bound complexes are aligned (Section 2.5). In principle, it is beneficial to refine independently each of the candidate solutions from an exhaustive rotational scanning, however this is computationally expensive and not feasible for large scale subtomogram classifications, which is necessary in whole cell tomography. We therefore provide also a stochastic parallel refinement framework (Section 2.3) to efficiently reduce the total number of refinement steps.

### Parameter definitions

For simplicity, we denote two subtomograms as two integrable functions *f*, *g *: ℝ^3 ^→ ℝ. For **a **∈ ℝ^3 ^, let *τ*_**a **_be the translation operator (*τ*_**a**_*g*)(**x**) := *g*(**x **- **a**). For a rotation *R *in the three-dimensional rotational group *SO*(3), let Λ_*R *_be the rotation operator, such that (Λ_*Rg*_)(**x**) := *g*[*R*^-1^(**x**)]. *R *can be represented as a 3 by 3 rotation matrix **R**. In this case, (*τ*_**a**_Λ_*Rg*_)(**x**) = *g*(**R**^-1^(**x **- **a**)).

The rigid transformation parameters combine both rotation and translation and are expressed as *β *= (*R*, **a**) = (*φ*, *θ*, *ψ*, *a*_1_, *a*_2_, *a*_3_)^⊤^, where (*φ*, *θ*, *ψ*)^⊤ ^are Euler angles in the 'ZYZ' convention [29], with the rotation *R*, and translation parameters **a **= (*a*_1_,*a*_2_,*a*_3_)^⊤^. In addition, for simplicity, we denote the combined rigid transformation operator *κ*_*β *_:= *τ*_**a**_Λ_*R*_.

### Local optimization of subtomogram alignment based on a real space constrained dissimilarity score (RCS)

We now describe the gradient-guided refinement for the subtomogram alignment, given a coarse initial solution for *R *and **a**. The goal is to identify a local optimal solution given the current values of *R *and a as the starting parameters. To perform the alignment one must define a dissimilarity measure for the alignment of the two subtomograms. Besides the low resolution and SNR of subtomograms, distortions due to missing data (ie, the missing wedge effect) make subtomogram alignment challenging, and these effects must be explicitly considered in the alignment process.

To address this problem, Förster *et al *proposed a constrained correlation measure with missing wedge corrections [11]. It is based on a transform that eliminates the coefficients in the missing wedge region. Let $M:{\mathbb{R}}^{3}\to \left\{0,1\right\}$ be a missing wedge mask function. The missing wedge mask function  M defines for each subtomogram the valid and missing Fourier coefficients in Fourier space. For example, in single tilt electron tomography with tilt angle range ±*θ*, the constrained correlation can be defined as $M\left(\xi \right):=I_{\left(\left|\xi_{3}\right|\leq \left|\xi_{1}\right|\text{tan}\left(\theta \right)\right)}\left(\xi \right)$. Then for a given subtomogram *f *one can define a Fourier space constrained subtomogram function as

(1) $$f_{1}:=ℜ\left\{F^{-1}\left[\left(Ff\right)M\left(\Lambda_{R}M\right)\right]\right\}$$

, where  denotes the real part of a complex function, and  F is the Fourier transform operator, and $M\left(\Lambda_{R}M\right)$ ensures that only those Fourier coefficients are considered that are defined in both subtomograms, i.e. these Fourier coefficients are not part of the missing wedge regions in any of the two subtomograms. Correspondingly, a Fourier space constrained subtomogram function for the second subtomogram *g *is defined as

(2) $$g_{1}:=ℜ\left\{F^{-1}\left[\left(F_{\tau_{a}}\Lambda_{Rg},\right)M\left(\Lambda_{R}M\right)\right]\right\}$$

The normalized subtomogram transforms can be defined as $Nf:=\frac{f_{1}-\mu \left(f_{1}\right)}{\sqrt{\int {\left(f_{1}-\mu \left(f_{1}\right)\right)}^{2}}}$, and $N_{\kappa_{\beta }}g:=\frac{g_{1}-\mu (g_{1})}{\sqrt{\int {\left(g_{1}-\mu (g_{1})\right)}^{2}}}$,

where *μ *is the mean operator, defined as $\mu f=\frac{\int f\left(x\right)}{Sf}$, and Sf denotes the size of the subtomogram *f*. *μf *is therefore the average intensity value of subtomogram *f*.

Then the constrained correlation is calculated as

(3) $$c:=\int NfN_{\kappa_{\beta }}g$$

Because of the subtomogram normalization, this constrained correlation is equivalent to a constrained dissimilarity score:

(4) $$d_{\beta }^{F}:=\int {\left|Nf-N_{\kappa_{\beta }}g\right|}^{2}=2-2c$$

For a given initial guess of the rotation *R *(for instance one of the local minima in a rotational search) one can determine the corresponding best translation *τ*_**a **_that minimizes the distance criteria *d *efficiently using Fast Fourier Transform (FFT)). Given any Λ_*R *_and *τ*_**a**_, we seek to obtain an increment Λ_Δ*R *_and corresponding *τ*_Δ**a **_so that

(5) $$d_{\left(\Lambda_{\Delta R}\Lambda_{R},\tau_{\Delta a}\tau_{a}\right)}^{F}\leq d_{\left(\Lambda_{R},\tau_{a}\right)}^{F}$$

Since Nf is fixed with respect to *β*, we use the Levenberg-Marquardt algorithm [30] to obtain such increments. This algorithm converges very fast.

Let **x**_*j*_, *j *= 1... *n *be the locations of all *n *voxels in the grid of the subtomogram, then we have a discrete form of the constrained dissimilarity score

(6) $$\tilde{d_{\beta }^{F}}:=\underset{j1}{\sum }{\left[\left(Nf\right)\left(x_{j}\right)-\left(N_{\kappa_{\beta }}g\right)\left(x_{j}\right)\right]}^{2}$$

According to the Levenberg-Marquardt algorithm, Δ*β *= (Δ*R*, Δ**a**) can be obtained by computing

(7) $$\Delta \beta =\left[J^{⊤}J+\lambda \;\text{diag}{\left(J^{⊤}J\right)}^{-1}J^{⊤}\left(f-g_{\beta }\right)\right]$$

Here **f **and **g**_*β *_are the vector representations

(8) $$f={\left(\left(Nf\right)\left(x_{1}\right),...,\left(Nf\right)\left(x_{n}\right)\right)}^{⊤}$$

and

(9) $$g_{\beta }={\left(\left(N_{\kappa_{\beta }}g\right)\left(x_{1}\right),...,\left(N_{\kappa_{\beta }}g\right)\left(x_{n}\right)\right)}^{⊤}$$

**J **is the Jacobian matrix whose *j*th row is $\frac{\partial \left(N_{\beta },g\right)\left(x_{j}\right)}{\partial \beta }$, which is approximated by numerical differentiation; the operator diag(**E**) converts a matrix **E **to a diagonal matrix consisting of only diagonal elements of **E**; λ is a damping factor to control the rate of convergence.

The final result of this section provides the refined alignment parameters *R*_2 _= *R*_1 _+ Δ*R*_1 _and **a**_2 _= **a**_1 _+ Δ**a**_1 _given the initial parameter set *R*_1 _and **a**_1_. To perform a complete alignment refinement this process must be repeated iteratively until convergence is achieved (next section).

### Stochastic parallel refinement process

**Figure 1:**
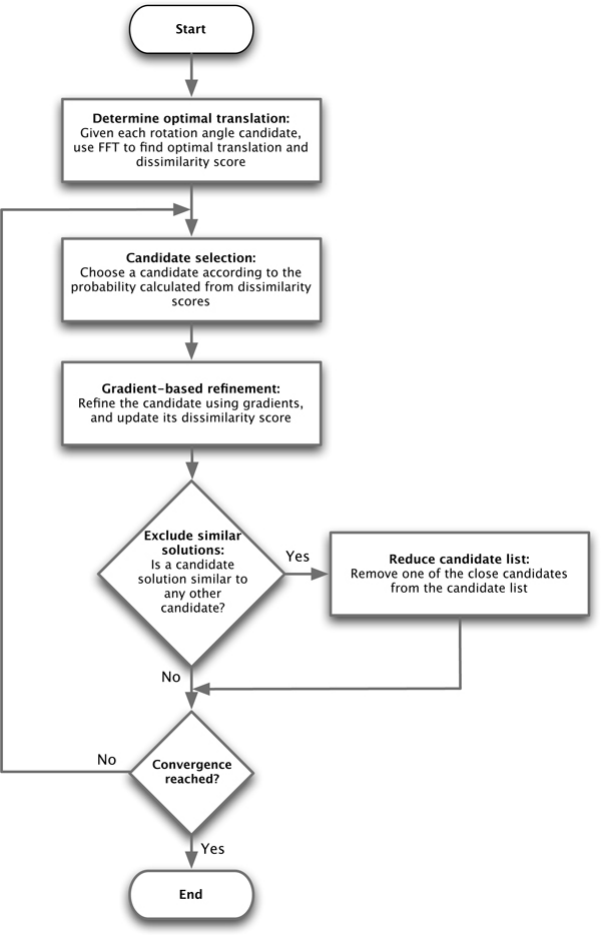
**Flow chart**. Flow chart of the stochastic parallel refinement process.

To carry out a global optimization it is necessary to perform multiple refinement runs starting each time from a different candidate rotation angle. However, to carry out these individual optimizations independently is time consuming, which would prevent large-scale applications of subtomogram alignments. Therefore, we propose a stochastic parallel refinement framework to prioritize for those candidate transform parameters with smaller dissimilarity scores (Figure 1). The basic idea of this iterative algorithm is to store the scores of all *m *candidate transformation parameters *β*_1_,..., *β*_*m*_, where each *β *= (*R*, **a**) consists of both rotation and translation parameters. The choice of which *β*_*j *_to refine next is stochastically decided according to a probability obtained from $d_{\beta_{j}}$. In other words, at each iteration candidate angles *β*_*j *_with smaller $d_{\beta_{j}}$ have a higher probability of being selected for refinement using the incremental method described in section 2.2.

We define a sampling probability that considers both rank and magnitude of *d*. Suppose the candidate parameters are ordered such that

(10) $$d_{\beta_{1}}\geq ...\geq d_{\beta_{m}}$$

Then for *j *= 1... *m *the sampling probability of *β*_*j *_is proportional to *p*_*j *_with

(11) $$p_{j}=p_{j-1}\text{max}\left(10^{t/\left(m-1\right)},d_{\beta_{j-1}}/d_{\beta_{j}}\right),\forall j=2...m$$

where *p*_1 _= 1 and *t *is a scaling threshold such that the distinction between *p*_*j *_and *p*_*j*-1 _is at least 10^*t*/(*m*-1)^, and *p*_*m*_/*p*_1 _≥ 10^*t*^.

To further enhance the computational efficiency, similar candidate transforms *β *are removed from the list to omit redundant optimization runs. The similarity of two transforms *β*_*j *_and *β*_*k *_is defined as the Frobenius norm ${∥D_{\beta_{j}\beta_{k}}∥}_{F}$, where

(12) $$D_{\beta_{j}\beta_{k}}:=\left[R_{j}^{-1}\left(I-\left(a_{j},a_{j},a_{j}\right)\right)\right]-\left[R_{k}^{-1}\left(I-\left(a_{k},a_{k},a_{k}\right)\right)\right]$$

∀*j*, *k *= 1,..., *m*. If ${∥D_{\beta_{j}\beta_{k}}∥}_{F}\leq \gamma$ is lower than a predefined threshold *γ*, then the transform leading to the larger of the two dissimilarity scores *d *is removed from the target list.

To terminate the optimization process, at each iteration the ratio between the smallest and the initial minimum score is calculated. The iterative process is terminated when convergence is achieved, which in turn is identified by a linear regression ratio *t*^regress ^over the minimal scores in the last iterations. In case convergence cannot be achieved the optimization is terminated after a large number of iterations *n*^max_iter^.

Similar to other stochastic optimization methods, such as genetic algorithms, our method also stores and evolves a population of candidate solutions. However, our method represents solutions by continuous values, and improves individual solutions by gradually refining them. By contrast, genetic algorithms usually encode solutions in strings of discrete bits, and generate new solutions by applying mutation and recombination on multiple existing solutions.

In this section we have introduced a parallel iterative refinement method that relies on a dissimilarity measure and local optimization process as described in Section 2.2. In the following section, we introduce another refinement method based on a different dissimilarity measure between subtomograms.

### Local optimization of subtomogram alignment based on a Fourier space constrained subtomogram dissimilarity score (FCS)

After having introduced an iterative refinement process, and introduced a dissimilarity measure in Section 2.2, we now test the refinement process further with a second dissimilarity score. This new score is based on a constrained dissimilarity score computed directly in Fourier space [14]:

(13) $$d_{\beta }^{\text{B}}:=\frac{\int {\left|\left(Ff\right)-\left(F_{\tau_{a}}\Lambda_{R}g\right)\right|}^{2}M\left(\Lambda_{R}M\right)}{\int M\left(\Lambda_{R}M\right)}$$

By properties of the Fourier transform

(14) $$\left(F_{\tau_{a}}\Lambda_{R}g\right)\left(\xi \right)=e^{-2\pi a^{⊤}\xi }\left(\Lambda_{R}Fg\right)\left(\xi \right)$$

, given a fixed initial *R*, the initial a can be efficiently calculated using FFT. Because $d_{\beta }^{B}$ is not expressed as the summed square of differences, here the Levenberg-Marquardt algorithm cannot be directly applied. However, because $\int M\left(\Lambda_{R}M\right)$ has a regular structure containing only binary 0 and 1 values, one can approximate $d_{\beta }^{B}$ as

(15) $$d_{\beta }^{B}\approx c\int {\left|\left(Ff\right)-\left(F_{\tau_{a}}\Lambda_{Rg}\right)\right|}^{2}M\left(\Lambda_{R}M\right)$$

where $c:=\frac{1}{\int M\left(\Lambda_{R}M\right)}$ is treated as a constant in the whole refinement step.

Let *ξ*_*j*_, *j *= 1... *n *be the locations of all *n *voxels in the grid of the Fourier transform of the tomogram such that $\left[M\left(\Lambda_{R}M\right)\right]\left(\xi_{j}\right)=1$. Then a discrete form of the dissimilarity score can be formulated as

(16) $$\tilde{d_{\beta }^{B}}:=c\underset{j}{\sum }{\left|\left(Ff\right)\left(\xi_{j}\right)-\left(F_{\kappa_{\beta }g}\right)\left(\xi_{j}\right)\right|}^{2}$$

Because the above score is based on complex functions, the Levenberg-Marquardt algorithm cannot be directly applied. Therefore we derive a new version of the Levenberg-Marquardt algorithm for complex functions. In this version, Δ*β *can be obtained by computing

(17) $$\Delta \beta =A^{-1}b$$

where

(18) $$A=\left[ℜ{\left(J\right)}^{⊤}ℜ\left(J\right)+픍{\left(J\right)}^{⊤}픍\left(J\right)\right]+\lambda \;\text{diag}\left[ℜ{\left(J\right)}^{⊤}ℜ\left(J\right)+픍{\left(J\right)}^{⊤}픍\left(J\right)\right]$$

and where  and  denote real and imaginary parts and

(19) $$b=ℜ{\left(J\right)}^{⊤}\left[ℜ\left(f\right)-ℜ\left(g_{\beta }\right)\right]+픍{\left(J\right)}^{⊤}\left[픍\left(f\right)-픍\left(g_{\beta }\right)\right]$$

Here **f **and **g**_*β *_are vector representations of the Fourier transform of the two subtomograms

(20) $$f={\left(\left(Ff\right)\left(\xi_{1}\right),...,\left(Ff\right)\left(\xi_{n}\right)\right)}^{⊤}$$

and

(21) $$g_{\beta }={\left(\left({ℱ}_{\kappa_{\beta }g}\right)\left(\xi_{1}\right),...,\left({ℱ}_{\kappa_{\beta }g}\right)\left(\xi_{n}\right)\right)}^{⊤}$$

**J **is the Jacobian matrix whose *j*th row is $\frac{\partial \left(F_{\kappa_{\beta }g}\right)\left(\xi_{j}\right)}{\partial \beta }$, where the derivative with respect to the translation parameters can be determined analytically (according to Equation (14)) and the derivative with respect to the rotation parameters is approximated by numerical differentiation. λ is a damping factor to control convergence speed.

In summary, in this section a Fourier-based similarity score is introduced and combined with a Levenberg-Marquardt algorithm adapted for complex functions.

### Constrained rotational search around a rotation axis

If knowledge about the macromolecule's preferred orientation is available, it is beneficial to reduce the rotational search space to a range of only those preferred orientations. Then a significantly smaller number of rigid candidate transformations is sufficient to find the optimal alignment. For example, when the macro-molecules are membrane-bound protein complexes (e.g. [7,27]), the feasible search is often constrained to rotations around an axis, which is the membrane surface normal at the position where the complex is attached to the membrane. In such a case, both subtomograms *f *and *g *can first be rotated so that their membrane surface normal are aligned (i.e., they are rotated to the direction that is parallel to z-axis). Then the alignment search is reduced to rotations of *g *around the z-axis in combination with a full translational search to minimize the dissimilarity score.

To minimize distortions due to the interpolation step in rigid transformations, one wants to reduce the number of sequential transformations for the original subtomograms. Therefore, we perform the constrained search by rotating only *g *using Λ_*R *_while keeping the original subtomogram *f *fixed. This procedure consists of three components:

(22) $$\Lambda_{R}={\left(\Lambda_{R_{f}}\right)}^{-1}\Lambda_{R_{n}}\Lambda_{R_{g}}$$

where *R*_*f *_and *R*_*g *_are the rotations of *f *and *g *so that the membrane surface normal are parallel to the z-axis. *R*_*n *_represents a rotation around the z-axis, defined in the form of (*φ*, 0,0)^⊤^. During the refinement process, *R*_*f *_and *R*_*g *_are kept constant, and the only rotational parameter to be optimized is *φ*, which is the rotation around the z-axis.

### Generating simulated cryo-electron tomograms

For a reliable assessment of the method, tomograms must be simulated as realistic as possible. We follow a previously applied methodology for realistically simulating the tomographic image formation [4,6,11,31].

Initial density maps at 4 nm resolution are generated and used as samples for simulating electron micrograph images at different tilt angles. The tilt angles are set within a certain maximal range with steps of 1°. As a result our data contains a wedge-shaped region in Fourier space for which no data has been measured (missing wedge effects), similar to experimental measurements. The missing wedge effect leads to distortions of the density maps in real spaces. To generate realistic micrographs, noise is added to the images and the resulting image map is convoluted with a Contrast Transfer Function (CTF), which describes the imaging in the transmission electron microscope in a linear approximation. Any negative contrast values beyond the first zero of the CTF are eliminated. We also consider the modulation Transfer Function (MTF) of a typical detector used in whole cell tomography, and convolute the density map with the corresponding MTF. The CTF and MTF describe distortions from interactions between electrons and the specimen and distortions due to the image detector [31,32]. Typical acquisition parameters used during actual experimental measurements of whole cell tomograms [4] were used: voxel spacing = 1 nm, the spherical aberration = 2 × 10^-3^m, the defocus value = -4 × 10^-6^m, the MTF corresponded to a realistic electron detector [33], defined as sinc(*πω*/2) where *ω *is the fraction of the Nyquist frequency.

**Figure 2:**
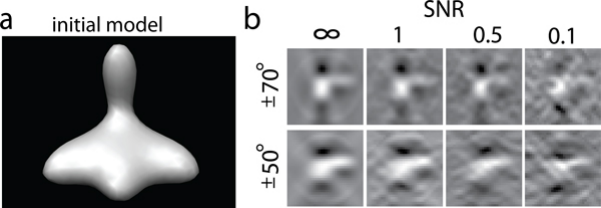
**Simulated subtomograms from phantom model**. (a) Density map of an unsymmetric phantom model consisting of four different 3D Gaussian functions. This density map is used to simulate subtomograms of 32^3 ^voxels. (b) A slice of the reconstructed tomograms at different levels of noise (∞, 1, 0.5, 0.1), and different tilt angle ranges leading to different levels of missing wedge distortions. The isosurface are generated using the Chimera software package [35]. The slices are plotted using MATLAB.

Finally, we use a backprojection reconstruction algorithm to generate a tomogram from the individual 2D micrographs that were generated at the various tilt angles [4]. To test the influence of increasing noise, we add different amount of noise to the images, so that the SNRs range between ∞ and 0.1, respectively. Figure 2(b) shows the reconstructed subtomograms of a phantom model at different noise levels and different tilt angle ranges.

All our methods are implemented in MATLAB.

## Results

We test our methods on phantom models and actual structures of protein complexes.

### Pairwise alignment of subtomograms from phantom models

To assess the general performance, 100 pairs of subtomograms with randomly placed phantom models were generated for different SNR levels and tilt angle ranges (Figure 2(b)). Our stochastic parallel refinement method is tested using both the RCS and FCS dissimilarity scores. We use following termination criterion for the optimization: *t*^regress ^≤ 0.001 and *n*^max_iter ^= 1000. We test our approach with respect to two factors. First, the average alignment error obtained from the refinement and second, the number of iterative steps that are needed to determine the optimal solution.

We show that even at a low SNR level of 0.5 and a typical range of tilt angles between -70° and +70° our method can still achieve a very low alignment error (Table 1). For example even when the rotational sampling is performed at only 60° intervals the stochastic iterative refinement process together with the RCS scoring produces on average errors of 3.1°, while the FCS scoring achieves 2.9° error (Table 1). This angle error is significantly lower than would be expected from exhaustive scanning where sampling of rotational angles is usually performed at 10° or 5° sampling intervals without additional refinement. When the rotational sampling is performed at 45° intervals, the performance is marginally improved to 2.7° (Table 1), indicating that the 60° interval is already sufficient for most alignment refinements.

**Table 1:** **Alignment rotation error**. Subtomogram alignment error in terms of the difference in the determined and true rotational angle of the subtomograms. Shown are the medians and median absolute deviations of all 100 subtomogram alignments. Bold font shows all the alignments with errors larger than 5°, which are considered inaccurate.

60° angle interval									
	SNR	∞	1	0.5	0.1	∞	1	0.5	0.1

Tilt									

±90°		0.71 ± 0.49	3.3 ± 2.8	2.6 ± 1.4	**14 ± 9.3**	0.89 ± 0.54	2.6 ± 2.1	2.4 ± 1.1	**8.5 ± 4.5**
±80°		0.85 ± 0.54	2.5 ± 1.8	3.5 ± 2.4	**21 ± 14**	1.1 ± 0.61	2.2 ± 1.6	3.2 ± 2.2	**12 ± 7.7**
±70°		1.2 ± 0.53	1.9 ± 1.3	3.1 ± 1.7	**19 ± 12**	2 ± 0.86	2.1 ± 1	2.9 ± 1.3	**16 ± 11**
±60°		0.97 ± 0.49	2 ± 0.97	3.7 ± 2.4	**49 ± 45**	1.5 ± 0.82	2.4 ± 1.2	3.8 ± 2.1	**34 ± 30**
±50°		1.8 ± 0.9	2.9 ± 1.6	**7 ± 5.2**	**87 ± 63**	2.6 ± 1.1	3.4 ± 1.8	**6.3 ± 4.2**	**43 ± 37**
±40°		1.6 ± 1	**9 ± 8.3**	**55 ± 53**	**123 ± 31**	**15 ± 14**	**92 ± 40**	**106 ± 37**	**113 ± 26**

45° angle interval									

	SNR	∞	1	0.5	0.1	∞	1	0.5	1

Tilt									

±90°		0.58 ± 0.25	1.1 ± 0.57	2 ± 0.84	**8.1 ± 2.7**	0.7 ± 0.34	1.1 ± 0.47	1.7 ± 0.77	**5.9 ± 2.4**
±80°		0.79 ± 0.31	1.4 ± 0.6	2.4 ± 0.93	**11 ± 4.5**	1.2 ± 0.49	1.7 ± 0.73	2.3 ± 0.97	**7.9 ± 3**
±70°		1 ± 0.26	1.8 ± 0.69	2.7 ± 1.2	**8.4 ± 3.1**	1.5 ± 0.46	2.1 ± 0.7	2.5 ± 1.1	**7.9 ± 2.5**
±60°		1 ± 0.42	1.6 ± 0.66	2.7 ± 0.86	**10 ± 4.8**	1.7 ± 0.68	2.1 ± 0.84	2.8 ± 0.84	**9.2 ± 4.5**
±50°		2 ± 0.77	2.4 ± 0.93	2.7 ± 1	**14 ± 11**	2.6 ± 1.1	2.9 ± 1.1	2.9 ± 1.1	**11 ± 5.1**
±40°		1.5 ± 0.79	2.5 ± 1.1	**5.7 ± 3.6**	**107 ± 27**	**9.4 ± 7.8**	**5.7 ± 3.3**	**7.7 ± 5.4**	**111 ± 19**

		RCS				FCS			

Our method therefore allows substantially larger sampling interval while maintaining a high accuracy in subtomogram alignment.

Using a sampling angle interval as large as 60° has major advantages in terms of computational efficiency. For the standard exhaustive scanning at 5° intervals a total of 168,634 candidate orientations must be processed while at 60° rotational intervals only 108 candidate orientations are refined. Also our method can in general achieve a small error for the translation of subtomograms that cannot be reached by an FFT based exhaustive sampling, which on average cannot be less than 0.5 (Table 2).

**Table 2:** **Alignment translation error**. Subtomogram alignment error in terms of the difference in the Euclidean distance between determined and true subtomogram translations. Shown are the medians and median absolute deviations of all 100 subtomogram alignments.

60° angle interval, RCS					
	SNR	∞	1	0.5	0.1

Tilt					

±90°		0.035 ± 0.023	0.16 ± 0.12	0.19 ± 0.12	0.96 ± 0.66
±80°		0.045 ± 0.029	0.24 ± 0.2	0.21 ± 0.15	1.3 ± 0.89
±70°		0.078 ± 0.037	0.25 ± 0.17	0.3 ± 0.18	1.3 ± 0.74
±60°		0.068 ± 0.036	0.19 ± 0.12	0.43 ± 0.3	2.2 ± 1.3
±50°		0.14 ± 0.078	0.26 ± 0.17	0.65 ± 0.51	2.3 ± 1.3
±40°		0.15 ± 0.092	0.74 ± 0.64	1.7 ± 1.3	3.2 ± 1.6

60° angle interval, FCS					

	SNR	∞	1	0.5	0.1

Tilt					

±90°		0.047 ± 0.023	0.12 ± 0.081	0.11 ± 0.053	0.49 ± 0.31
±80°		0.053 ± 0.03	0.15 ± 0.1	0.18 ± 0.1	0.85 ± 0.66
±70°		0.11 ± 0.057	0.13 ± 0.074	0.21 ± 0.1	0.95 ± 0.58
±60°		0.11 ± 0.061	0.2 ± 0.094	0.3 ± 0.15	1.6 ± 1.2
±50°		0.19 ± 0.1	0.28 ± 0.16	0.44 ± 0.26	1.8 ± 1.2
±40°		0.61 ± 0.54	3.3 ± 2.7	4.3 ± 2.6	6.2 ± 3

45° angle interval, RCS					

	SNR	∞	1	0.5	0.1

Tilt					

±90°		0.031 ± 0.014	0.072 ± 0.031	0.12 ± 0.049	0.43 ± 0.21
±80°		0.051 ± 0.027	0.11 ± 0.051	0.17 ± 0.072	0.69 ± 0.32
±70°		0.063 ± 0.024	0.14 ± 0.052	0.21 ± 0.1	0.63 ± 0.24
±60°		0.076 ± 0.036	0.15 ± 0.068	0.23 ± 0.1	0.89 ± 0.5
±50°		0.11 ± 0.055	0.2 ± 0.094	0.28 ± 0.14	1.3 ± 0.95
±40°		0.14 ± 0.071	0.31 ± 0.17	0.67 ± 0.47	6.2 ± 5.3

45° angle interval, FCS					

	SNR	∞	1	0.5	0.1

Tilt					

±90°		0.033 ± 0.016	0.071 ± 0.03	0.094 ± 0.032	0.29 ± 0.13
±80°		0.061 ± 0.031	0.1 ± 0.052	0.13 ± 0.062	0.46 ± 0.21
±70°		0.08 ± 0.04	0.12 ± 0.05	0.17 ± 0.075	0.49 ± 0.22
±60°		0.1 ± 0.052	0.17 ± 0.091	0.22 ± 0.073	0.72 ± 0.36
±50°		0.19 ± 0.094	0.22 ± 0.083	0.24 ± 0.12	0.93 ± 0.5
±40°		0.76 ± 0.64	0.51 ± 0.36	0.82 ± 0.6	9.8 ± 4.1

**Figure 3:**
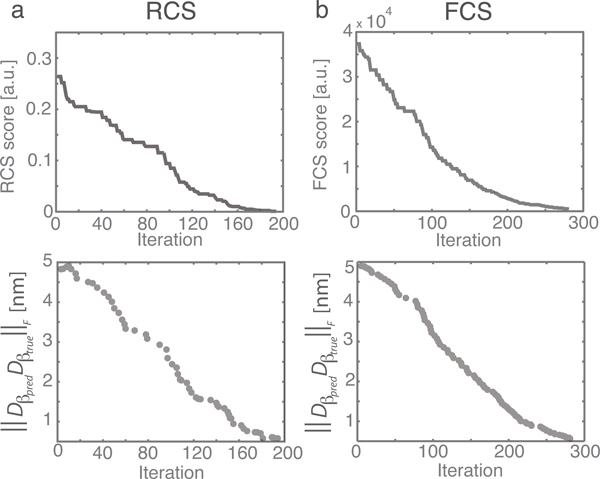
**Convergence example**. Top panels: The minimum dissimilarity scores obtained at different iterations subtracted from the true dissimilarity score. Bottom panels: The difference ${∥D_{\beta_{pred}\beta_{true}}∥}_{F}$ between predicted and true transforms at those iterations where minimum dissimilarity scores are obtained. Left, subtomogram alignments based on the real space constrained dissimilarity score (RCS). Right, alignment based on the Fourier space constrained dissimilarity score (FCS). Shown is the performance for subtomograms with SNR 0.5, tilt angle range ±60°. The method was tested with rotation angle seeds sampled at angle interval 60°.

**Figure 4:**
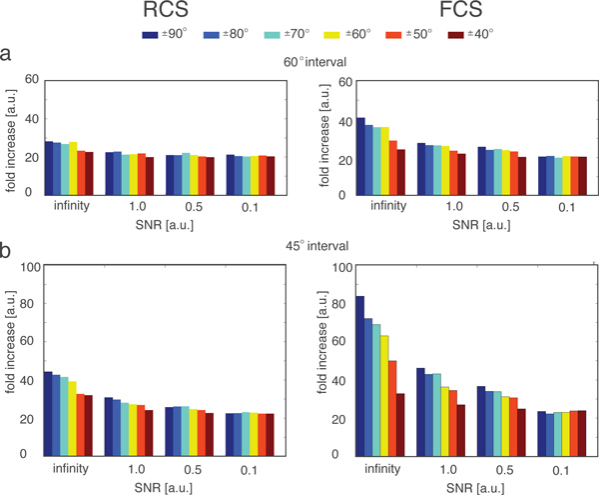
**Computation speedup**. Computational speed up of the stochastic parallel optimization method compared to the traditional exhaustive refinement method. Shown is the ratio of the number of iterations needed to find the optimal solution for the exhaustive and stochastic parallel optimization methods (The numbers show the fold increase in number of iterations when the exhaustive method is used). Shown are the median deviations of all 100 subtomogram alignments for the RCS method (left column) and FCS method (right column) for optimizations using a rotational search at 60° intervals (a) and 45° intervals (b), respectively.

In addition, the parallel stochastic refinement process reduces considerably the number of refinement iterations that are needed to reach a good solution in an optimization. At a rotational sampling of 60°, there are 108 candidate orientations that can potentially serve as starting points for a refinement process. Without the parallel stochastic optimization method, a refinement of a candidate orientation takes on average about 60 iterations per run. When all candidate orientations are refined independently a total of about 6480 iterative refinement steps are needed to find the global optimum among all candidate orientations. However, our parallel stochastic refinement process reaches convergence already within only 200-300 iterative refinement steps (Figure 3). We estimate that the parallel stochastic refinement is on average about 20 to 40 fold faster in comparison to the independent refinement of all candidate orientations (Figure 4). At a rotational sampling of 45°, the speedup leads to an 84 fold faster alignment (Figure 4).

Next, we test the alignment when the search space is constrained to rotations around a single axis. When rotational sampling is performed at 60° and 45° intervals, only 6 and 8 initial candidate rotation angles are used, respectively. The alignment performance is shown in Tables 3 and 4.

**Table 3:** **Constrained alignment rotation error**. Constraining the search to rotations around a single axis. Subtomogram alignment error in terms of the difference in the determined and true rotational angle. Shown are the medians and median absolute deviations of all 100 subtomogram alignments. Bold font shows all the alignments with errors larger than 5°, which are considered inaccurate.

60° angle interval									
	SNR	∞	1	0.5	0.1	∞	1	0.5	0.1
Tilt									

±90°		0.2 ± 0.14	0.31 ± 0.15	0.55 ± 0.26	4.1 ± 1.8	0.21 ± 0.13	0.44 ± 0.24	0.62 ± 0.33	3.1 ± 1.7
±80°		0.29 ± 0.19	0.55 ± 0.38	0.89 ± 0.62	4.5 ± 3	0.44 ± 0.29	0.67 ± 0.41	1 ± 0.73	3.2 ± 1.6
±70°		0.43 ± 0.25	0.67 ± 0.38	0.81 ± 0.54	**5.4 ± 3.9**	0.57 ± 0.37	0.85 ± 0.53	0.84 ± 0.43	3.8 ± 2.4
±60°		0.6 ± 0.4	0.99 ± 0.79	1.5 ± 1.2	**5.9 ± 4.9**	0.81 ± 0.64	1.3 ± 1.1	1.7 ± 1.3	3.8 ± 3
±50°		1.1 ± 0.93	1.3 ± 1.1	1.4 ± 0.94	**5 ± 4.1**	1.7 ± 1.4	1.7 ± 1.4	2 ± 1.3	4.1 ± 3.6
±40°		1.7 ± 1.7	2.3 ± 2.2	**7 ± 6.9**	**42 ± 38**	3.9 ± 3.7	3.1 ± 2.9	4 ± 3.6	**42 ± 39**

45° angle interval									

	SNR	∞	1	0.5	0.1	∞	1	0.5	0.1

Tilt									

±90°		0.2 ± 0.12	0.35 ± 0.16	0.42 ± 0.25	3.1 ± 1.7	0.19 ± 0.12	0.38 ± 0.19	0.45 ± 0.28	2.5 ± 1.2
±80°		0.18 ± 0.12	0.31 ± 0.21	0.61 ± 0.3	3.9 ± 1.7	0.35 ± 0.23	0.5 ± 0.34	0.5 ± 0.36	2.5 ± 1.4
±70°		0.28 ± 0.15	0.47 ± 0.29	0.56 ± 0.31	3.9 ± 2.1	0.5 ± 0.34	0.64 ± 0.43	0.63 ± 0.45	2.8 ± 1.7
±60°		0.43 ± 0.23	0.49 ± 0.28	0.72 ± 0.37	4.5 ± 3	0.67 ± 0.46	0.64 ± 0.39	0.92 ± 0.46	2.7 ± 1.7
±50°		0.65 ± 0.41	0.89 ± 0.53	0.93 ± 0.64	**5.2 ± 3.4**	0.99 ± 0.67	1.1 ± 0.72	1.1 ± 0.81	3.1 ± 2.2
±40°		0.98 ± 0.87	1.2 ± 0.9	2 ± 1.7	**12 ± 11**	1.6 ± 1.2	1.7 ± 1.3	1.6 ± 1.2	**9.6 ± 9.2**

		RCS				FCS			

**Table 4:** **Constrained alignment translation error**. Constraining the search to rotations around a single axis. Subtomogram alignment error in terms of the difference in the Euclidean distance between determined and true subtomogram translations. Shown are the medians and median absolute deviations of all 100 subtomogram alignments.

60° angle interval, RCS					
	SNR	∞	1	0.5	1

Tilt					

±90°		0.018 ± 0.0053	0.047 ± 0.017	0.08 ± 0.023	0.28 ± 0.1
±80°		0.027 ± 0.011	0.069 ± 0.029	0.1 ± 0.048	0.37 ± 0.17
±70°		0.037 ± 0.017	0.085 ± 0.04	0.13 ± 0.059	0.45 ± 0.28
± 60°		0.055 ± 0.028	0.14 ± 0.083	0.19 ± 0.11	0.59 ± 0.36
±50°		0.1 ± 0.067	0.18 ± 0.12	0.24 ± 0.12	0.74 ± 0.42
±40°		0.27 ± 0.25	0.49 ± 0.4	0.94 ± 0.83	3.3 ± 2.3

60° angle interval, FCS					

	SNR	∞	1	0.5	0.1

Tilt					

±90°		0.018 ± 0.0062	0.05 ± 0.017	0.074 ± 0.021	0.23 ± 0.097
±80°		0.027 ± 0.013	0.063 ± 0.025	0.076 ± 0.029	0.24 ± 0.091
±70°		0.034 ± 0.015	0.077 ± 0.032	0.098 ± 0.036	0.32 ± 0.16
±60°		0.055 ± 0.033	0.12 ± 0.063	0.18 ± 0.099	0.46 ± 0.28
±50°		0.11 ± 0.075	0.16 ± 0.099	0.2 ± 0.11	0.6 ± 0.36
±40°		0.39 ± 0.35	0.36 ± 0.27	0.57 ± 0.44	3.5 ± 2.8

45° angle interval, RCS					

	SNR	∞	1	0.5	0.1

Tilt					

±90°		0.015 ± 0.005	0.054 ± 0.014	0.063 ± 0.019	0.24 ± 0.086
±80°		0.024 ± 0.0072	0.056 ± 0.018	0.086 ± 0.028	0.29 ± 0.12
±70°		0.033 ± 0.0095	0.075 ± 0.028	0.11 ± 0.04	0.38 ± 0.17
±60°		0.046 ± 0.019	0.1 ± 0.036	0.16 ± 0.051	0.48 ± 0.22
±50°		0.067 ± 0.035	0.14 ± 0.06	0.22 ± 0.098	0.59 ± 0.27
±40°		0.22 ± 0.18	0.31 ± 0.21	0.44 ± 0.29	2.3 ± 1.7

45° angle interval, FCS					

	SNR	∞	1	0.5	0.1

Tilt					

±90°		0.017 ± 0.0053	0.048 ± 0.014	0.06 ± 0.016	0.21 ± 0.074
±80°		0.021 ± 0.0069	0.052 ± 0.018	0.073 ± 0.025	0.2 ± 0.067
±70°		0.03 ± 0.011	0.065 ± 0.023	0.098 ± 0.03	0.26 ± 0.085
±60°		0.043 ± 0.017	0.088 ± 0.033	0.13 ± 0.044	0.32 ± 0.11
±50°		0.07 ± 0.032	0.14 ± 0.056	0.18 ± 0.073	0.44 ± 0.19
±40°		0.17 ± 0.11	0.24 ± 0.16	0.33 ± 0.18	1.5 ± 1.2

When the information about the orientation of the membrane surface normal is included in the search process, the alignment accuracy increases significantly for subtomograms at high distortion levels. Without surface normal information, the alignment fails for subtomograms at very low SNR of 0.1, resulting in average angluar alignment errors of at least 10°. With surface normal information, the average anglular alignment errors are less than 6° even for subtomograms generated from a small tilt angle range of ±50°.

**Figure 5:**
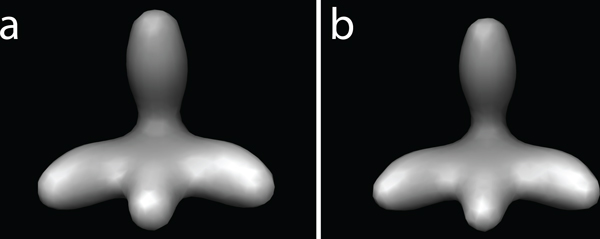
**Averaged subtomogram after alignment**. Averaged subtomograms. Left, aligned using RCS. Right, aligned using FCS.

Next, we further test our alignment methods for refining the density maps of the complexes by averaging over all aligned subtomograms. For each complex, we generated 1000 subtomograms (at SNR 0.5, tilt angle range ±60°) containing randomly oriented models. We then aligned the tomograms against the initial templates with a rotational sampling of 60° angle intervals. From the resulting averaged density maps it can be seen that our methods can successfully recover the initial structures (Figure 5).

### Pairwise alignment of subtomograms from real macromolecular complexes

A whole cell cryo-electron tomogram consists of instances of macromolecular complexes of different types. In principle, these instances can be segmented into individual subtomograms and classified after pairwise alignments. Therefore, subtomogram alignment and classification is fundamental for successful structural systems biology analysis of complexes using whole cell tomograms. In this section, we test our methods on subtomograms of four macromolecular complexes obtained from the Protein Data Bank (PDB id 1KP8, 2GHO, 1W6T, 1YG6). The density map of each complex is calculated from its atomic structure by applying a low pass filter at 4 nm resolution using the PDB2VOL program of the *Situs 2.0 *package [34] and voxel spacing of 1 nm. The resulting density maps are used to simulate 20 subtomograms for each randomly placed macromolecular complex, at SNR 0.5 and tilt angle range ±60° (Section 2.6).

**Figure 6:**
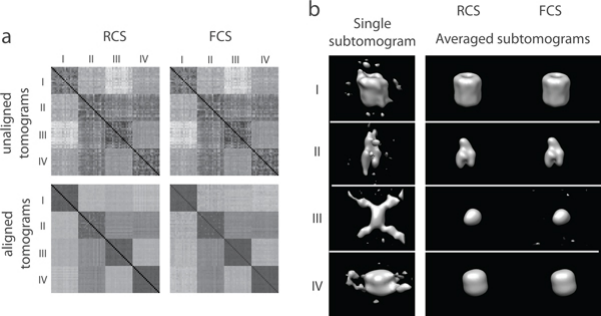
**Pairwise alignment of protein complexes**. (a) Dissimilarity score matrices for subtomogram classification. The matrix elements representing the same complexes are in consecutive order. (Top row) Dissimilarity score matrix based on the initial subtomogram orientations before alignment for (left column) RCS score and (right column) FCS score. (Bottom row) RCS and FCS score matrices after subtomogram alignments. The alignment is performed at a sampling with 60° rotation angle intervals. (b) Density maps of complexes generated after averaging of the aligned subtomograms in the same class. (Left column) isosurface of the density distribution in single subtomogram for each complex. (Middle and right columns) isosurface of the resulting density maps generated by averaging the 20 subtomograms aligned with the RCS and FCS scores, respectively.

We perform all pairwise alignments between all 80 subtomograms with sampling of 60° rotational angle intervals. After alignment the resulting dissimilarity score matrix for subtomogram classification is significantly improved in comparison to the dissimilarity score matrix generated from the initial starting structures (Figure 6(a)).

After classification and alignment, the resulting averaged tomograms are very similar to the original density maps. The distortions, as evident in the individual subtomograms are greatly reduced after averaging (Figure 6(b)).

## Conclusion

In this paper, we have proposed a new gradient-based method for high precision subtomogram alignments. Combined with the RCS and FCS scores, this method can achieve significantly lower alignment errors in comparison to an exhaustive sampling method. We show that this accuracy can already be reached with only a relatively small number of sampled candidate orientations, for example at rotational intervals of 60° and 45°. The improvement in performance when using rotational intervals of 45° instead of 45° intervals is only marginal, indicating that 60° intervals are already sufficient for most alignments. We further extended the method to a special case when the alignment search is constrained to rotations around a single axis. For instance, alignment of membrane bound complexes allow the rotational search to be restricted to rotations around an axis parallel to a surface normal. This constrained alignment can achieve even higher alignment precision and is more robust to distortions in subtomograms, even when only 6 to 8 initial rotation angle candidates are used.

The RCS and FCS scores both have certain advantages. In contrast to FCS the RCS score takes into account the contrast difference between subtomograms. On the other hand, the FCS score has closed form partial derivatives with respect to the translation parameters, therefore introducing less numerical instability in the gradient refinement process. Moreover it is more efficiently computed because a smaller number of computational intensive rigid transform operations are needed.

Moreover, we have proposed a very efficient stochastic parallel refinement method, which is able to find the global optimum with only a small fraction of iterations in comparison to the independent sampling and refinement with the same sampling angle intervals. Together, these improvements increase significantly the efficiency and accuracy for subtomogram alignments, which is a key factor for the systematic classification of macromolecular complexes in cryo-electron tomograms of whole cells.

## Competing interests

The authors declare that they have no competing interests.

## Authors' contributions

F.A. and M.X conceived the project. M.X. performed the research and carried out experiments. F.A. and M.X. wrote the manuscript.

## References

[B1] Kühner S Van Noort V Betts M Leo-Macias A Batisse C Rode M Yamada T Maier T Bader S Beltran-Alvarez P Proteome organization in a genome-reduced bacterium Science 2009 326 5957 1235 10.1126/science.1176343 19965468

[B2] Gehlenborg N O'Donoghue S Baliga N Goesmann A Hibbs M Kitano H Kohlbacher O Neuweger H Schneider R Tenenbaum D Visualization of omics data for systems biology Nat Methods 2010 7 3 Suppl S56 68 20195258 10.1038/nmeth.1436

[B3] Nickell S Kofler C Leis A Baumeister W A visual approach to proteomics Nature reviews Molecular cell biology 2006 7 3 225 230 10.1038/nrm1861 16482091

[B4] Beck M Malmström J Lange V Schmidt A Deutsch E Aebersold R Visual proteomics of the human pathogen Leptospira interrogans Nature methods 2009 6 11 817 823 10.1038/nmeth.1390 19838170 PMC2862215

[B5] Beck M Topf M Frazier Z Tjong H Xu M Zhang S Alber F Exploring the Spatial and Temporal Organization of a Cell's Proteome Journal of Structural Biology 2011 173 3 483 496 10.1016/j.jsb.2010.11.011 21094684 PMC3784337

[B6] Xu M Beck M Alber F Template-free detection of macromolecular complexes in cryo electron tomograms Bioinformatics 2011 27 13 i69 i76 10.1093/bioinformatics/btr207 21685103 PMC3117359

[B7] Avinoam O Fridman K Valansi C Abutbul I Zeev-Ben-Mordehai T Maurer U Sapir A Danino D Grünewald K White J Conserved Eukaryotic Fusogens Can Fuse Viral Envelopes to Cells Science 2011 332 6029 589 10.1126/science.1202333 21436398 PMC3084904

[B8] Briegel A Ortega D Tocheva E Wuichet K Li Z Chen S Müller A Iancu C Murphy G Dobro M Universal architecture of bacterial chemoreceptor arrays Proc Natl Acad Sci U S A 2009 106 40 17181 17186 10.1073/pnas.0905181106 19805102 PMC2761316

[B9] Morris D Jensen G Toward a biomechanical understanding of whole bacterial cells Annu Rev Biochem 2008 77 583 613 10.1146/annurev.biochem.77.061206.173846 18355161

[B10] Zitova B Flusser J Image registration methods: a survey Image and vision computing 2003 21 11 977 1000 10.1016/S0262-8856(03)00137-9

[B11] Förster F Pruggnaller S Seybert A Frangakis A Classification of cryo-electron sub-tomograms using constrained correlation Journal of structural biology 2008 161 3 276 286 10.1016/j.jsb.2007.07.006 17720536

[B12] Amat F Comolli L Moussavi F Smit J Downing K Horowitz M Subtomogram alignment by adaptive Fourier coefficient thresholding Journal of structural biology 2010 171 3 332 344 10.1016/j.jsb.2010.05.013 20621702 PMC4189811

[B13] Hrabe T Chen Y Pfeffer S Kuhn Cuellar L Mangold A Förster F PyTom: A python-based toolbox for localization of macromolecules in cryo-electron tomograms and subtomogram analysis Journal of structural biology 2012 178 2 177 188 10.1016/j.jsb.2011.12.003 22193517

[B14] Bartesaghi A Sprechmann P Liu J Randall G Sapiro G Subramaniam S Classification and 3D averaging with missing wedge correction in biological electron tomography Journal of structural biology 2008 162 3 436 450 10.1016/j.jsb.2008.02.008 18440828 PMC2556382

[B15] Xu M Beck M Alber F High-throughput subtomogram alignment and classification by Fourier space constrained fast volumetric matching Journal of structural biology 2012 178 2 152 164 10.1016/j.jsb.2012.02.014 22420977 PMC3821800

[B16] Volkmann N Methods for segmentation and interpretation of electron tomographic reconstructions Methods Enzymol 2010 483 31 46 20888468 10.1016/S0076-6879(10)83002-2

[B17] Schmid M Booth C Methods for aligning and for averaging 3D volumes with missing data Journal of structural biology 2008 161 3 243 248 10.1016/j.jsb.2007.09.018 18299206 PMC2680136

[B18] Walz J Typke D Nitsch M Koster A Hegerl R Baumeister W Electron tomography of single ice-embedded macromolecules: three-dimensional alignment and classification Journal of structural biology 1997 120 3 387 395 10.1006/jsbi.1997.3934 9441941

[B19] Winkler H 3D reconstruction and processing of volumetric data incryo-electron tomography Journal of structural biology 2007 157 1 126 137 10.1016/j.jsb.2006.07.014 16973379

[B20] Heumann JM Hoenger A Mastronarde DN Clustering and variance mapsfor cryo-electron tomography using wedge-masked differences Journal of structural biology 2011 175 3 288 299 10.1016/j.jsb.2011.05.011 21616153 PMC3150390

[B21] Scheres SH Melero R Valle M Carazo JM Averaging of electron subtomograms and random conical tilt reconstructions through likelihood optimization Structure 2009 17 12 1563 1572 10.1016/j.str.2009.10.009 20004160 PMC2940245

[B22] Stolken M Beck F Haller T Hegerl R Gutsche I Carazo JM Baumeister W Scheres SH Nickell S Maximum likelihood based classification of electron tomographic data Journal of structural biology 2010 173 1 77 85 20719249 10.1016/j.jsb.2010.08.005

[B23] Winkler H Zhu P Liu J Ye F Roux KH Taylor KA Tomographic subvolume alignment and subvolume classification applied to myosin V and SIV envelope spikes Journal of structural biology 2009 165 2 64 77 10.1016/j.jsb.2008.10.004 19032983 PMC2656979

[B24] Yu L Snapp RR Ruiz T Radermacher M Probabilistic principal component analysis with expectation maximization (PPCA-EM) facilitates volume classification and estimates the missing data Journal of structural biology 2010 171 1 18 30 10.1016/j.jsb.2010.04.002 20385241 PMC3353830

[B25] Yu Z Frangakis AS Classification of electron sub-tomograms with neural networks and its application to template-matching Journal of structural biology 2011 174 3 494 504 10.1016/j.jsb.2011.02.009 21382496

[B26] Castaño-Díez D Kudryashev M Arheit M Stahlberg H Dynamo A flexible, user-friendly development tool for subtomogram averaging of cryo-EM data in High-Performance Computing Journal of structural biology 2012 178 2 139 151 10.1016/j.jsb.2011.12.017 22245546

[B27] Förster F Medalia O Zauberman N Baumeister W Fass D Retrovirus envelope protein complex structure in situ studied by cryo-electron tomography Proceedings of the National Academy of Sciences of the United States of America 2005 102 13 4729 10.1073/pnas.0409178102 15774580 PMC555690

[B28] Martin Beck V Snapshots of nuclear pore complexes in action captured by cryo-electron tomography Nature 2007 449 7162 611 615 10.1038/nature06170 17851530

[B29] Brink D Satchler G Angular momentum 1993 Oxford University Press, USA

[B30] Nocedal J Wright S Numerical optimization 2006 Springer, Berlin

[B31] Nickell S Förster F Linaroudis A Net W Beck F Hegerl R Baumeister W Plitzko J TOM software toolbox: acquisition and analysis for electron tomography Journal of Structural Biology 2005 149 3 227 234 10.1016/j.jsb.2004.10.006 15721576

[B32] Frank J Three-dimensional electron microscopy of macromolecular assemblies: visualization of biological molecules in their native state 2006 Oxford University Press, USA

[B33] McMullan G Chen S Henderson R Faruqi A Detective quantum efficiency of electron area detectors in electron microscopy Ultramicroscopy 2009 109 9 1126 1143 10.1016/j.ultramic.2009.04.002 19497671 PMC2864625

[B34] Wriggers W Milligan R McCammon J Situs: A Package for Docking Crystal Structures into Low-Resolution Maps from Electron Microscopy Journal of Structural Biology 1999 125 2-3 185 195 10.1006/jsbi.1998.4080 10222274

[B35] Pettersen E Goddard T Huang C Couch G Greenblatt D Meng E Ferrin T UCSF Chimera-a visualization system for exploratory research and analysis Journal of computational chemistry 2004 25 13 1605 1612 10.1002/jcc.20084 15264254

